# Mutagenesis of seed storage protein genes in *Soybean* using CRISPR/Cas9

**DOI:** 10.1186/s13104-019-4207-2

**Published:** 2019-03-27

**Authors:** Chenlong Li, Vi Nguyen, Jun Liu, Wenqun Fu, Chen Chen, Kangfu Yu, Yuhai Cui

**Affiliations:** 1 London Research and Development Center, Agriculture and Agri-Food Canada, London, ON Canada; 2 Department of Biology, Western University, London, ON Canada; 3 State Key Laboratory of Biocontrol and Guangdong Key Laboratory of Plant Resource, School of Life Sciences, Sun Yat-sen University, Guangzhou, China; 4 Guangdong Academy of Agricultural Sciences, Guangzhou, China; 5 Department of Biological Science and Technology, Minnan Normal University, Zhangzhou, China; 6 Agriculture and Agri-Food Canada, Harrow Research and Development Centre, Harrow, ON Canada

**Keywords:** Seed storage proteins, Soybean, CRISPR/Cas9, Genome editing

## Abstract

**Objective:**

Soybean seeds are an important source of vegetable proteins for both food and industry worldwide. Conglycinins (7S) and glycinins (11S), which are two major families of storage proteins encoded by a small family of genes, account for about 70% of total soy seed protein. Mutant alleles of these genes are often necessary in certain breeding programs, as the relative abundance of these protein subunits affect amino acid composition and soy food properties. In this study, we set out to test the efficiency of the CRISPR/Cas9 system in editing soybean storage protein genes using *Agrobacterium rhizogenes*-mediated hairy root transformation system.

**Results:**

We designed and tested sgRNAs to target nine different major storage protein genes and detected DNA mutations in three storage protein genes in soybean hairy roots, at a ratio ranging from 3.8 to 43.7%. Our work provides a useful resource for future soybean breeders to engineer/develop varieties with mutations in seed storage proteins.

**Electronic supplementary material:**

The online version of this article (10.1186/s13104-019-4207-2) contains supplementary material, which is available to authorized users.

## Introduction

Just as seeds are an important foundation of Agriculture, soybean seeds are an important source of vegetable proteins for both food and industry world-wide. Two major families of storage proteins, conglycinins (7S) and glycinins (11S), account for about 70% of total soy seed protein [1]. Both quantity and quality of storage proteins, in soybean seeds, are major biochemical components influencing the quality of tofu and other soy food products [2, 3]. Soybean breeders have developed a series of mutant soybean genotypes differing in seed storage glycinin and conglycinin subunit composition. These mutant lines encompass a wide range of genetic variability available to breeders to improve soy protein functional properties for specific end uses [4–6]. Conventionally, breeders have to repeatedly introgress the mutations into elite soybean cultivars by conducting genetic crosses and rounds of selection over several generations and years. This is a long and labour-intensive process, which has been a major limiting factor for the timely delivery of quality soybean varieties, in an effort to cope with a continuously changing agriculture environment. Even though new plant breeding techniques have been constantly sought after by the plant genetics and genomics research community, it seems that the CRISPR-Cas9 system (the Clustered Regularly Interspaced Short Palindromic Repeat (CRISPR)/CRISPR-associated 9 (Cas9)) is revolutionizing our breeding practices [7–9].

The CRISPR/Cas9 system has emerged as a robust technology for efficient genome editing [10, 11], and has been successfully applied in many major crops, including soybean [12–16]. In this study, we set out to test the efficiency of the CRISPR/Cas9 system in editing soybean storage protein genes using *Agrobacterium rhizogenes*-mediated hairy root transformation system. Since stable transgenic soybean plants require a relatively long time (approximately 9 months) to develop, we opted to use soybean hairy roots as a model system which only takes about 3 weeks. Therefore, assessing the efficiency of sgRNAs in generating InDels at target sites in hairy roots, prior to whole-plant transformation, could solve the labor-intensive problem of the traditional transformation techniques. More importantly, soybean hairy roots are true soybean tissue and thus, making them ideal for purposes of quick testing of the genome editing efficiency of the sgRNAs and method optimization.

## Main text

### Materials and methods

#### Plant materials and growth conditions

Wild-type soybean line “Harosoy 63” was gifted from Dr. Sangeeta Dhaubhadel. “Harosoy 63” was registered in 1964 [17]. All pots and trays were sterilized with 10% bleach solution. Seeds were sterilized with H_2_O_2_/ethanol (10% of 30% H_2_O_2_, 75% of 95% ethanol, 15% sterile distilled water) for 2 min by inverting gently. Washing solution was decanted and seeds were rinsed 5–6 times with excess amount of sterile distilled water. Pots were filled with sterilized vermiculite and sterilized seeds were placed 1–2 cm deep in vermiculite.

#### SgRNA Design and Construction of sgRNA: Cas9 Expression

We used EnsemblPlants (http://plants.ensembl.org/Glycine_max/Gene/Summary?g=GLYMA_19G254600;r=19:49937807-49940339;t=KRG97147;db=core) to obtain the gene sequences for all nine conglycinin and glycinin genes. To design sgRNA, we employed CRISPR-PLANT (https://www.genome.arizona.edu/crispr/CRISPRsearch.html). Sequences of all sgRNAs are listed in Table 1. We used *pZG23C05* vector (Cas9/gRNA construction kit for dicots, Bar resistant from ZGene Biotechnology Inc.) and followed the manufacturer’s protocol to construct our gRNA target sequences into Cas9/gRNA plasmid. Plants and bacteria transformed by *pZG23C05* should be resistant to Basta and Kanamycin, respectively.

**Table 1:** Summary of mutations generated for each sgRNA

Genes	ID for sgRNA	gRNA-PAM^a^	No. hairy roots genotyped	No. hairy roots w. mutations detected	% mutation
*Glyma.20g148400*	g1	5′-CCTTCTGATGAGGTGGGCGT**GGG**-3′	17	1	5.8
*Glyma.20g146200*	g1	5′-GGACAATCCGGTAGTCTCGA**AGG**-3′	46	0	
	g2	5′-ACAGAAGCAGAAACAGGAAG**AGG**-3′	37	0	
	g3	5′-GGATTCTCTGGGCATCGCCA**CCC**-3′	42	0	
*Glyma.10g246300*	g1	5′-TCTCGCTATTGCAACTTCGG**AGG**-3′	27	0	
	g2	5′-CAGTGTTGTGGATATGAACG**AGG**-3′	32	0	
	g3	5′-AGAAGAAGAAGACCAAGACG**AGG**-3′	21	0	
*Glyma.20g148200*	g1	5′-GGATTCTCTGGGCATCGCCA**GGG**-3′	75	0	
	g2	5′-ACTCTCTTTGAGAACCAAAA**CGG**-3′	63	0	
	g3	5′-GGACAATCCGGTAGTCTCGA**AGG**-3′	28	0	
*Glyma.10g037100*	g1	5′-CATCTTACTCACCTTATCCC**CGG**-3′	99	0	
	g2	5′-GACGTACTCGTGATTCCTC**CGG**-3′	54	0	
	g3	5′-CTCCATTCGCGGCTCTTGCG**AGG**-3′	42	0	
*Glyma.03g163500*	g1	5′-GATAACCGTATAGAGTCAGA**AGG**-3′	26	1	3.8
*Glyma.19g164900*	g1	5′-GATAACCGTATAGAGTCAGA**AGG**-3′	32	14	43.7
*Glyma.13g123500*	g1	5′-GACCACCGCGTTGAGTCCGA**AGG**-3′	38	0	
	g2	5′-GACGTACTAGTGATTCCTCC**TGG**-3′	64	0	
	g3	5′-TCGGGCCTGCTTGGTCGCTG**TGG**-3′	48	0	
*Glyma.19g164800*	g1	5′-GACAACCTCATCGAATCCCA**AGG**-3′	49	0	
	g2	5′-AGCGTGGCCTACGTGACGAG**TGG**-3′	76	0	
	g3	5′-TCGGTGTTCAGCGGCGCTGT**TGG**-3′	49	0	

^a^The PAM sequences are highlighted in bold

#### Hairy root transformation using *A. rhizogenes* K599

The plasmid vector used for expressing Cas9 and single-guide RNA was mobilized into *A. rhizogenes* K599 via electroporation. Hairy root transformation was performed following [18]. The 5-day-old seedlings with unopened cotyledons were selected for inoculation. A syringe needle was used to deliver the bacterial culture by stabbing at the cotyledonary node and/or at the hypocotyl proximal to the cotyledon. The puncture site was covered with sterile moist vermiculite. The whole tray was covered with a clear dome and left in a growth cabinet at 28 °C/12 h light (200 µmol/m^2^s light intensity), 25 °C/12 h dark and 80% humidity.

#### Evaluation of the ratio of transgenic hairy roots

To evaluate the ratio of transgenic hairy roots, a Green Florescence Protein (GFP) expressing vector pB7WG2D [19] was used. Hairy roots grown to a length of 5–6 cm were labelled with numbers and screened using a dissecting fluorescence microscope (Nikon SMZ1500), and the transgenic roots showed bright GFP fluorescence labeling while non-transgenic roots remained dark.

#### Screening for mutations induced by the CRISPR/Cas9 system

Genomic DNA was extracted from hairy roots by adding 400 µl extraction buffer (3% CTAB, 4% B-Me EtOH, 2 M NaCl, 5% PVP, 100 mM Tris–HCl, 25 mM EDTA) and incubating for 45 min at 70 °C water bath. 400 µl of chloroform was added and quickly vortexed. The samples were spun at 14,000 rpm for 10 min. About 400 µl of the supernatant was aspirated into a new centrifuge tube and 400 µl of isopropanol was added to precipitate the DNA at − 20 °C for 30 min. The DNA was pelleted by spinning at 14,000 rpm for 20 min. DNA pellets were washed 2 times with 750 µl of 75% ethanol, spinning at 14,000 rpm each time. DNA pellets were dissolved with 50 µl of sterile distilled water. For PCR, 5 µl of DNA was used to amplify the genomic fragments containing the sgRNA targeting sites. PCR primers were designed to amplify a 400–800 bp amplicon containing the target sequence, which was cloned into pGEM^®^-T Easy vector (Promega). Bacterial colony PCR was conducted and positive clones were picked for sequencing. Primer sequences used are listed in Additional file 1: Table S1.

### Results and discussion

**Fig. 1:**
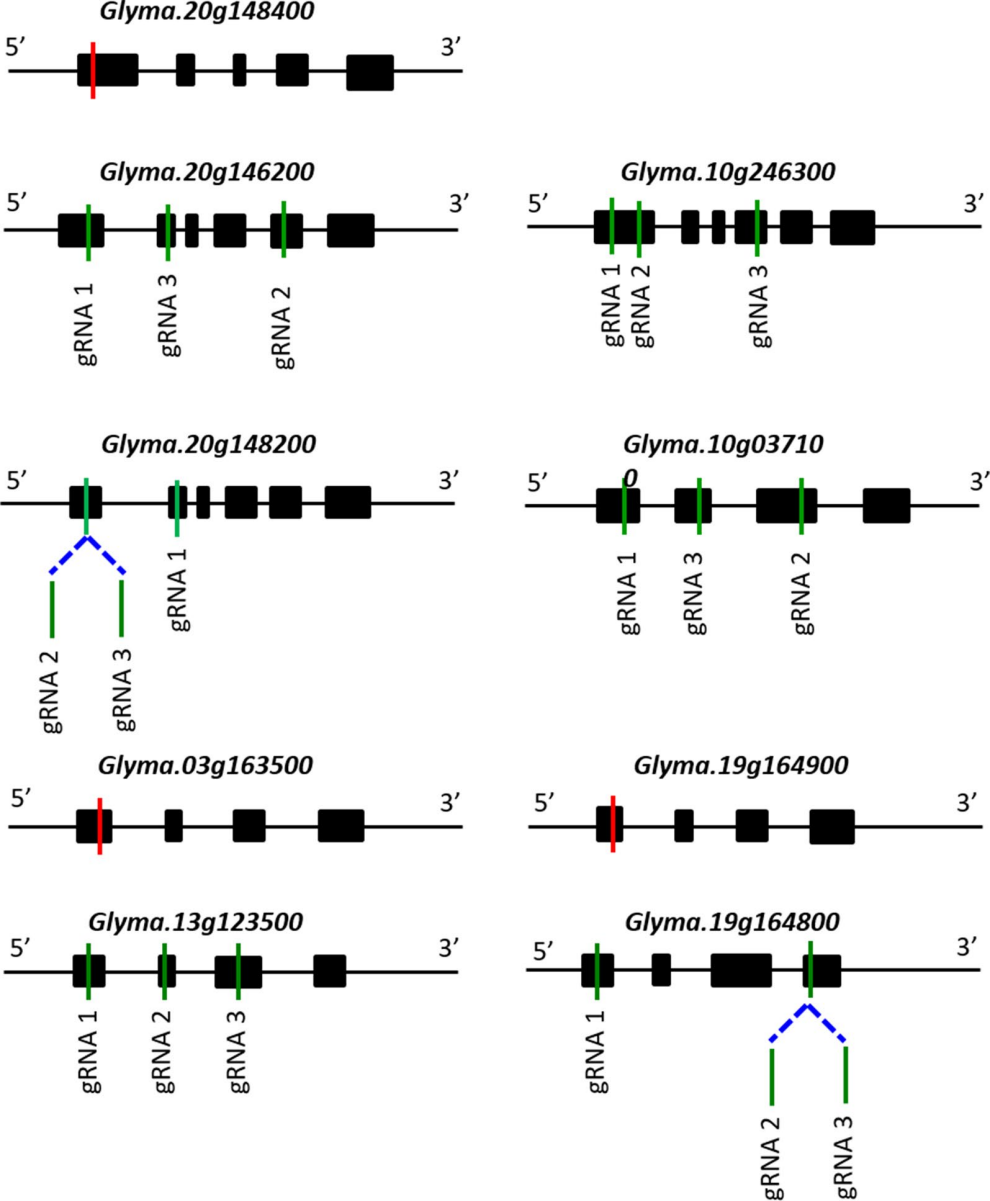
Schematic representation of nine storage protein genes and sgRNAs used in this study. Black boxes and lines represent exons and non-coding regions, respectively. Red and green vertical lines indicate sgRNAs that caused mutation and failed to make mutation, respectively

To explore whether the CRISPR/Cas9 system can generate mutations in soybean genes encoding seed storage proteins, we designed one sgRNA for each of the nine seed storage protein genes. Since our goal was to find sgRNAs that could disrupt the coding sequences, we tried to choose the sgRNA that targets the first exon of each gene. And when the first chosen sgRNA failed to cause any mutation, we continued to test additional up to three sgRNAs for each gene. Schematic presentations showing the positions of the sgRNAs in each gene are depicted in Fig. 1. The AGI numbers of the nine soybean storage protein genes are *Glyma.20g148400*, *Glyma.20g146200*, *Glyma.10g246300*, *Glyma.20g148200*, *Glyma.10g037100*, *Glyma.03g163500, Glyma.19g164900, Glyma.13g123500*, and *Glyma.19g164800*. The sgRNAs (driven by the *AtU6* promoter) were individually cloned into the *pZG23C05* vector carrying Basta (driven by the *35S* promoter) and *Cas9* (driven by the *Ubi* promoter) expression cassettes (Additional file 2: Figure S1).

The constructs were introduced into *Agrobacterium rhizogenes K599*, whose bacterial cultures were then used to inoculate at the junction site between the cotyledon and hypocotyl of 5-day-old soybean seedling to induce hairy roots (Additional file 3: Figure S2). About 10–15 days after inoculation, hairy roots could be observed to emerge from the puncture sites. Hairy roots were harvested individually at day 20 and genotyped for rapid evaluation of gene editing. Previous studies have shown that hairy roots induced by *Agrobacterium rhizogenes* may not necessarily be transgenic. Thus, in order to estimate the percentage of transgenic hairy roots in our transformation system, we used a construct that expresses Green Florescent Protein (GFP) under the control of the *35S* promoter. Positive transgenic roots can easily be distinguished from non-transgenic ones by checking the GFP signal under a microscope (Additional file 4: Figure S3). The ratio of GFP positive hairy roots differed substantially (0–80%) in each plant (Additional file 5: Table S2). However, among the 385 hairy roots generated from 54 soybean plants, 112 hairy roots were positive transgenic roots showing strong GFP signal. The average ratio of transgenic roots, in our hairy root transformation system, is therefore 29.1%.

**Fig. 2:**
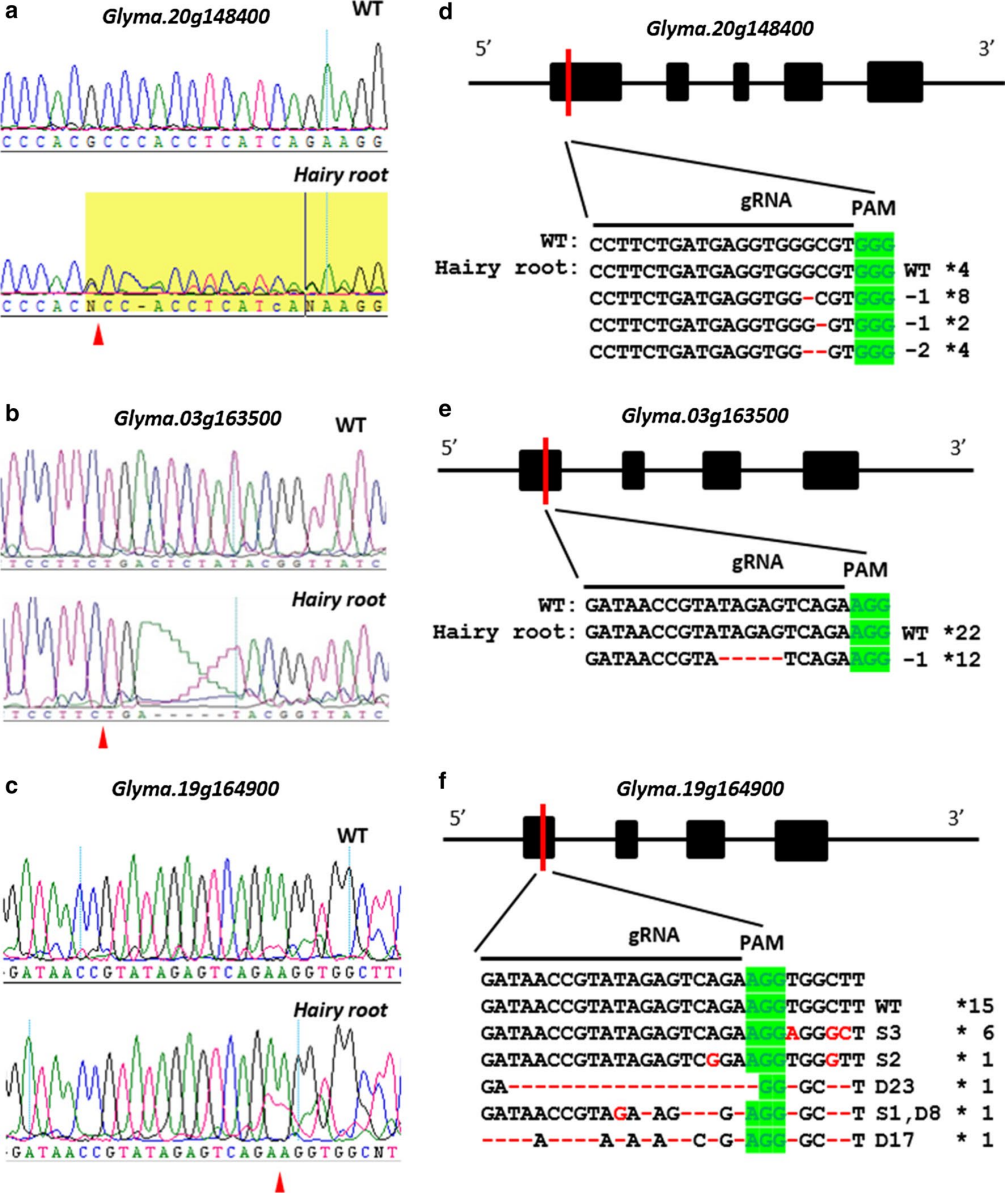
CRISPR/Cas9-mediated disruption of soybean storage protein genes in hairy roots. DNA sequencing peaks showing successful gene editing in target regions of *Glyma20g28650/Glyma20g28660* (**a**), *Glyma030g32030* (**b**), and *Glyma19g34780* (**c**). Sequencing result from WT served as the negative control. Red triangles point to the putative cutting sites by Cas9. Cloning and Sequencing results of mutant alleles of *Glyma20g28650/Glyma20g28660* (**d**), *Glyma030g32030* (**e**), and *Glyma19g34780* (**f**). The top row is the schematic representation of genomic locus. Black boxes and black lines represent exons and UTRs, respectively. Red vertical line indicates the position of sgRNAs. Letters D and S indicate the number of nucleotides deleted and substituted, respectively. The asterisks indicate the numbers of independent clones sequenced

In order to quickly evaluate gene editing efficacy of the sgRNAs in soybean storage protein genes, we directly sanger sequenced PCR products amplified from genomic DNAs of individual hairy roots. PCR primers were designed to warrant detection of the targeting sites of sgRNAs in the PCR products. In this analysis, we used at least 8 plants or 17 hairy roots for each gene. We reasoned that, if the editing efficacy is relatively high, the chromatograph of the sequencing results should show “mixed” peaks at the edited nucleotide(s). From the data collected, we found gene editing events in 3 out of 9 genes. First, we found that 1 out of 17 hairy roots showing gene editing by the sgRNA (5′-CCTTCTGATGAGGTGGGCGT-3′) which was predicted to target the first exon of gene *Glyma.20g148400* (Fig. 2a and Table 1). Second, for the sgRNA (5′-GATAACCGTATAGAGTCAGA-3′) that is predicted to target the first exon of both *Glyma.03g163500* and *Glyma.19g164900*, we indeed observed editing of both genes. In *Glyma.03g163500*, editing by this sgRNA was identified in 1 hairy root (26 roots tested), and editing in *Glyma.19g164900* was detected in 14 out of 32 hairy roots examined (Fig. 2b, c, and Table 1). These data suggest that two identical target sites in two different soybean storage protein genes could be simultaneously mutated by only one customized sgRNA. This might be beneficial for the application of CRIPSR/Cas9 to soybean when disruption of two genes is required at the same time. In terms of the editing efficiency, gene editing events were detected at about 5.8% of 17 hairy roots for *Glyma.20g148400*, 3.8% for *Glyma.03g163500*, and 43.7% for *Glyma.19g164900.* However, since the average ratio of transgenic hairy roots is not 100% as estimated above (Additional file 5: Table S2), the actual ratio of gene editing for these three genes could potentially be higher. For the remaining six sgRNAs which did not produce any editing, we designed and tested two more sgRNAs for each of them (Table 1). Despite our extensive screening of a large number of hairy roots, we were not able to detect signs of gene editing based on PCR-sanger sequencing. It has been previously reported that some genomic regions are more difficult to be edited by CRISPR/Cas9 [20–22], however, the precise reason(s) for this observation remain(s) largely unclear.

To investigate whether the editing events found in these 3 genes caused frame shifts, we cloned and sequenced the PCR fragments. Several different types of mutations were detected (Fig. 2d–f). For gene *Glyma.20g148400*, we observed one to two nucleotides deletions (Fig. 2d). In gene *Glyma.03g163500*, a 5-bp DNA deletion (missing TAGAG) was detected (Fig. 2e). And lastly, in gene *Glyma.19g164900,* we found five different types of InDels, some of which contain small deletion of up to 23 nucleotides (Fig. 2f). These PCR-sequencing results clearly demonstrate that our sgRNA constructs could cause mutations that would disrupt the reading frames of the seed storage protein genes. Since these target sites are all located upstream in the coding regions of the genes, these mutant alleles, when recapitulated in stable soybean transgenic plants, would be considered as null alleles, i.e., the storage protein subunits they encode would not be expressed/detected.

In conclusion, we have demonstrated that CRISPR/Cas9 system could mutate seed storage protein genes in soybean hairy roots. Different sgRNAs produced different types of mutations, and even the same sgRNA at two identical target sites but in two different loci could produce different InDels. Regarding those storage protein genes that were difficult candidates for editing by CRISPR, editing events might still be happening, but possibly with too low efficiency to be detected by sanger sequencing of the PCR products. This observation also strongly suggests that testing the effectiveness of sgRNAs at target sites, in soybean hairy roots, before generating transgenic plants can be time saving, less labor intensive and more cost effective. Our results confirm that the CRISPR system is a simple and inexpensive method that could be applied for editing seed storage protein genes in soybean, and that the sgRNAs reported in this work would be a useful resource for future soybean breeders to engineer/develop varieties containing new seed storage protein alleles for specific needs of certain breeding programs.

## Limitations

In this study, three out of nine soybean seed storage protein genes were edited by CRISPR/Cas9. One possible reason for the low efficiency could be that the CRISPR/Cas9 vector used in this study is not efficient enough for soybean genome editing. Further optimization of vectors might improve the editing efficiency. In addition, functional studies of those mutations on the seed storage proteins using stable soybean transgenic lines remain to be performed in this study.

## Additional files

**Additional file 1: Table S1.** Primers used in this study.

**Additional file 2: Figure S1.** Schematic representation of CRISPR/Cas9 vectors used in this study. The sgRNA, Basta resistant gene, and *Cas9* are driven by the *AtU6*-*26* promoter, *35S* promoter, and *Ubi* promoter, respectively.

**Additional file 3: Figure S2.** *Agrobacterium rhizogenes*-mediated induction of soybean hairy roots using CRISPR/Cas9 vectors. Junction site between cotyledon and hypocotyl, of 5-day-old soybean seedling, was inoculated to induce hairy roots. About 10–15 days after inoculation, hairy roots started emerging from puncture sites.

**Additional file 4: Figure S3.** Transformation efficiency of soybean hairy roots assessed by a GFP reporter construct. Constructs containing Green Florescent Protein (GFP), under the control of 35S promoter or empty vectors, were introduced into *Agrobacterium rhizogenes* to induce hairy roots. Positive transgenic roots, indicated by red arrows, can easily be distinguished from non-transgenic roots by checking the GFP signal by microscopy.

**Additional file 5: Table S2.** Summary of GFP-positive hairy roots at puncture sites.

## Abbreviations

7S: conglycinins
11S: glycinins
CRISPR-Cas9: the Clustered Regularly Interspaced Short Palindromic Repeat/CRISPR-associated 9
sgRNA: single-guide RNA
DSB: DNA double strand break
NHEJ: non-homologous end joining pathway
HDR: homology-directed repair
InDels: insertions and/or deletions
GFP: Green Florescent Protein
