# Supplementary material for: Mutagenesis of seed storage protein genes in Soybean using CRISPR/Cas9
Source: BMC Res Notes. 2019 Mar 27;12:176. doi: 10.1186/s13104-019-4207-2 (PMC6437971; doi:10.1186/s13104-019-4207-2)
Supplement: Supplementary file 1 — Additional file 1: Table S1. Primers used in this study. [file 13104_2019_4207_MOESM1_ESM.docx]

| Gene name | Forward primer 5’-3’ | Reverse primer 5’-3’ | sgRNA tested |
| --- | --- | --- | --- |
| *Glyma.20g148400* | AAGATGAGCAACCACGTCCA | TCAGCATGGTTGGGGAGAAG | g1 |
| *Glyma.20g146200* | TTGGTGTTGCTGGGAACTGT  CCCACAGCTTCGGGACTTG  ACTAGCCGTGACTGTTTTTATGT | GATTCTGGTGGTCGTGAGGG  CAATCAGGCACCAAAGAATTGGA  ACTGTTGGTTGCTTATTACATGG | g1  g2  g3 |
| *Glyma.10g246300* | TATGATGAGAGCGCGGTTCC  ACGTCCATTCCCATTCCCAC  CAGCAACAAGGGGAGGAGAG | GTGGGAATGGGAATGGACGT  GAAGCTGTTGGGAGCGTTTG  CCGCACTTCCAAAGGTTGCT | g1  g2  g3 |
| *Glyma.20g148200* | AGCCGTGACTGTTTTTATGTCC  TTGGTGTTGCTGGGAACTGT  TTGGTGTTGCTGGGAACTGT | ATCTGCCAGGTTTGTTGACG  ACATAAAAACAGTCACGGCTAGT  CGCCAGGGTGAAGGTTGTAG | g1  g2  g3 |
| *Glyma.10g03710* | CCTCTCATACCTATAAATCACCA  TGTGATTGTTTTGTTTGGTGATGA  TCAGCGTTATCAGCCCCAAG | TTTGAACAAGTGGTGACAAATGGA  TTGTTGCATGGTCTCTGGGT  AGGGTTGTAGAAGTCAGCGC | g1  g2  g3 |
| *Glyma.03g163500* | GCAAAACGAGTGCCAGATCC | TGAAGTTTGGACAAGATGGACC | g1 |
| *Glyma.19g164900* | CTGCTTTTCAGTGGCTGCTG | AGCGTTGGTGTAGGAAGGTC | g1 |
| *Glyma.13g123500* | TTCGTGTTCTCCACTTCACCAA  GCATTTCCGGGATGTCCTGA  TCCCACTCTGACAACTCCCA | TGAGTAAGATGGCAAGTGGAG  CAGTGACATGTGTATCACTGCT  CGAGTCACCGAAGTCGTTTG | g1  g2  g3 |
| *Glyma.19g164800* | CTTAGCACGACAGCAATGCC  GCTCTGCTTTTGCAGAGTGG  GCTCTGCTTTTGCAGAGTGG | TGAAGTTGTTGGATAACCTTGGAG  CTCAGCCCAAAAGCATTCGC  CTCAGCCCAAAAGCATTCGC | g1  g2  g3 |

**Additional Table S1. Primers used in this study**
