# Supplementary figures and images for: Mutagenesis of seed storage protein genes in Soybean using CRISPR/Cas9
Source: BMC Res Notes. 2019 Mar 27;12:176. doi: 10.1186/s13104-019-4207-2 (PMC6437971; doi:10.1186/s13104-019-4207-2)

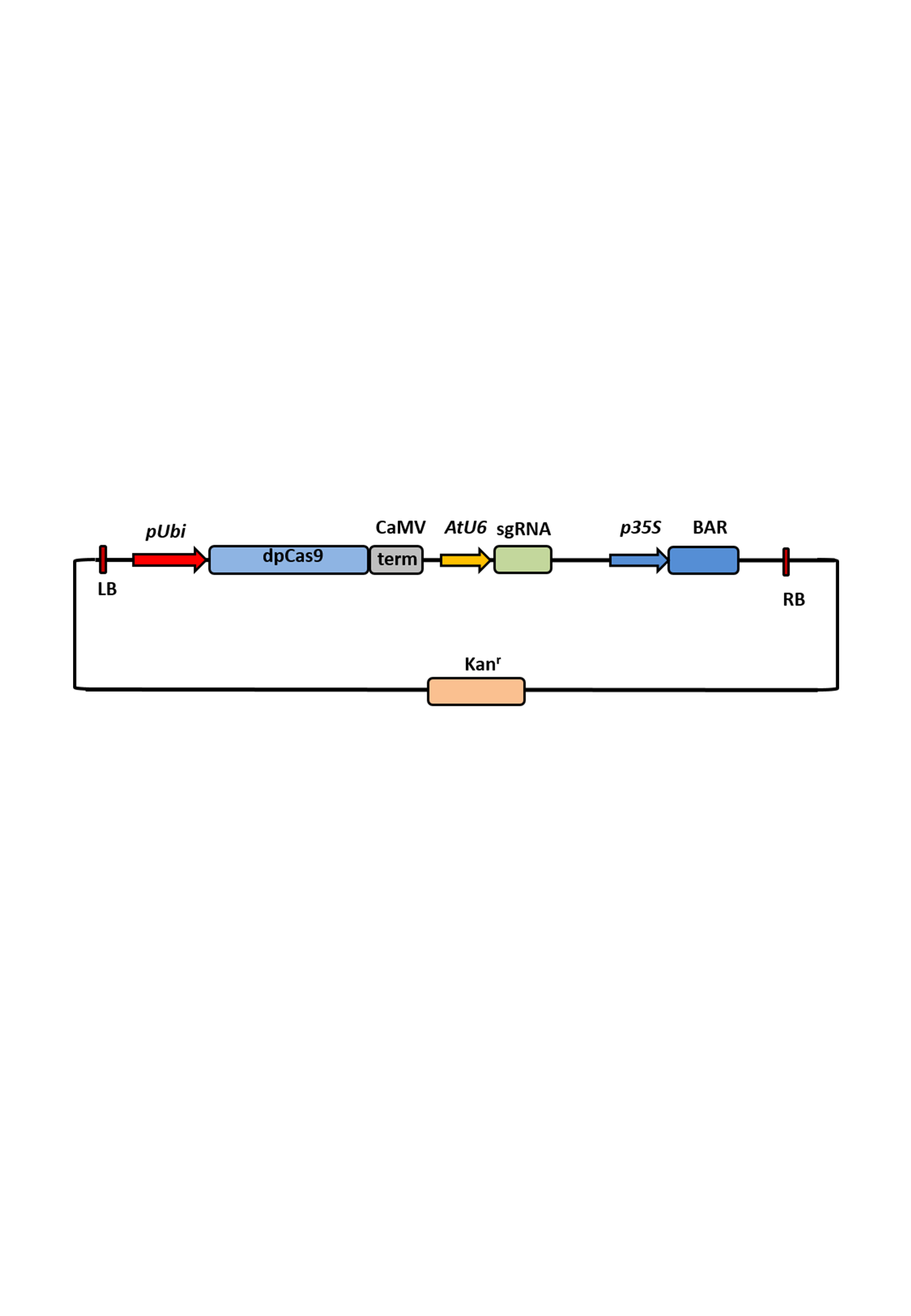

Supplement: Supplementary file 2 — Additional file 2: Figure S1. Schematic representation of CRISPR/Cas9 vectors used in this study. The sgRNA, Basta resistant gene, and Cas9 are driven by the AtU6-26 promoter, 35S promoter, and Ubi promoter, respectively. [file 13104_2019_4207_MOESM2_ESM.jpg]

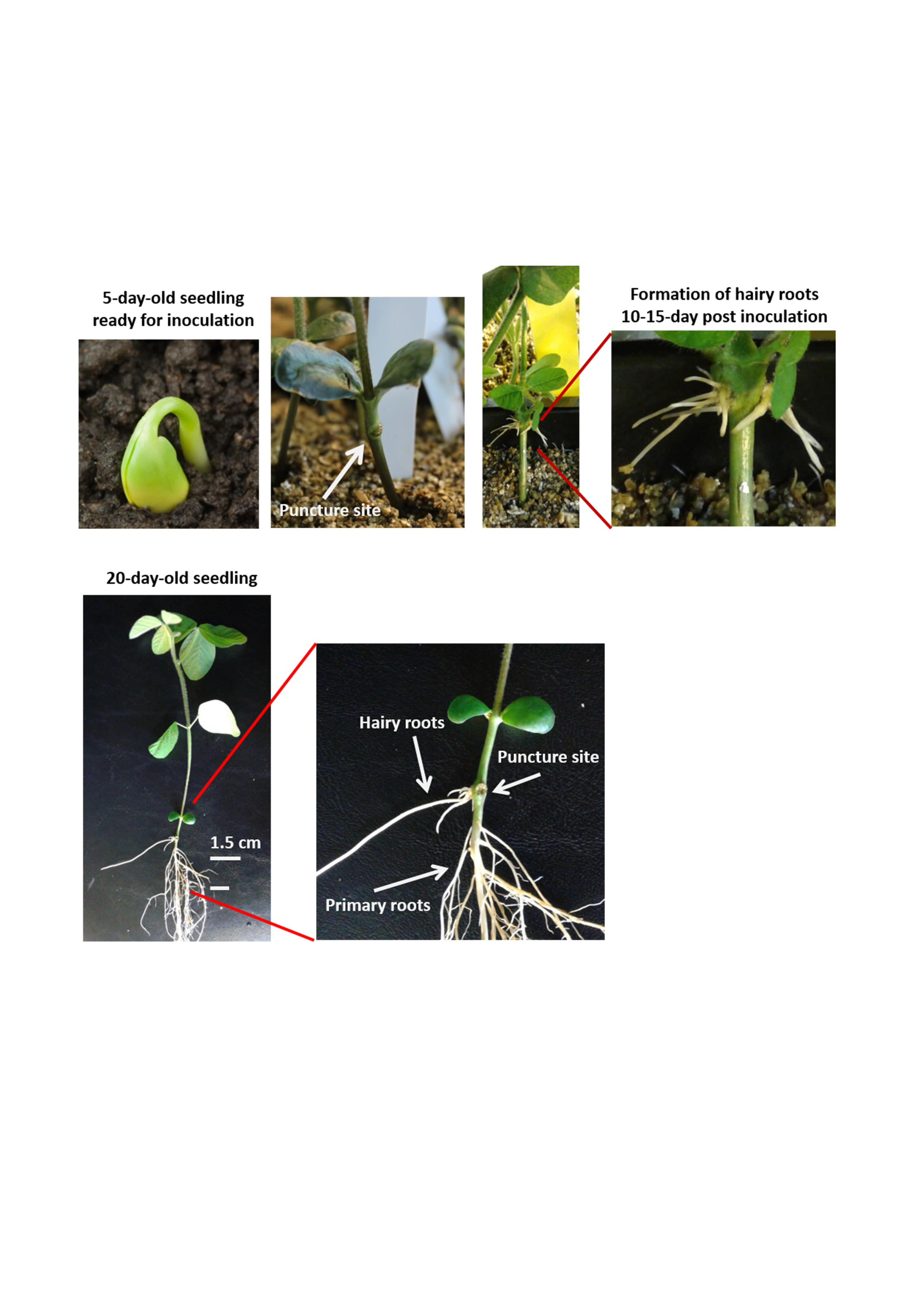

Supplement: Supplementary file 3 — Additional file 3: Figure S2. Agrobacterium rhizogenes-mediated induction of soybean hairy roots using CRISPR/Cas9 vectors. Junction site between cotyledon and hypocotyl, of 5-day-old soybean seedling, was inoculated to induce hairy roots. About 10–15 days after inoculation, hairy roots started emerging from puncture sites. [file 13104_2019_4207_MOESM3_ESM.tif]

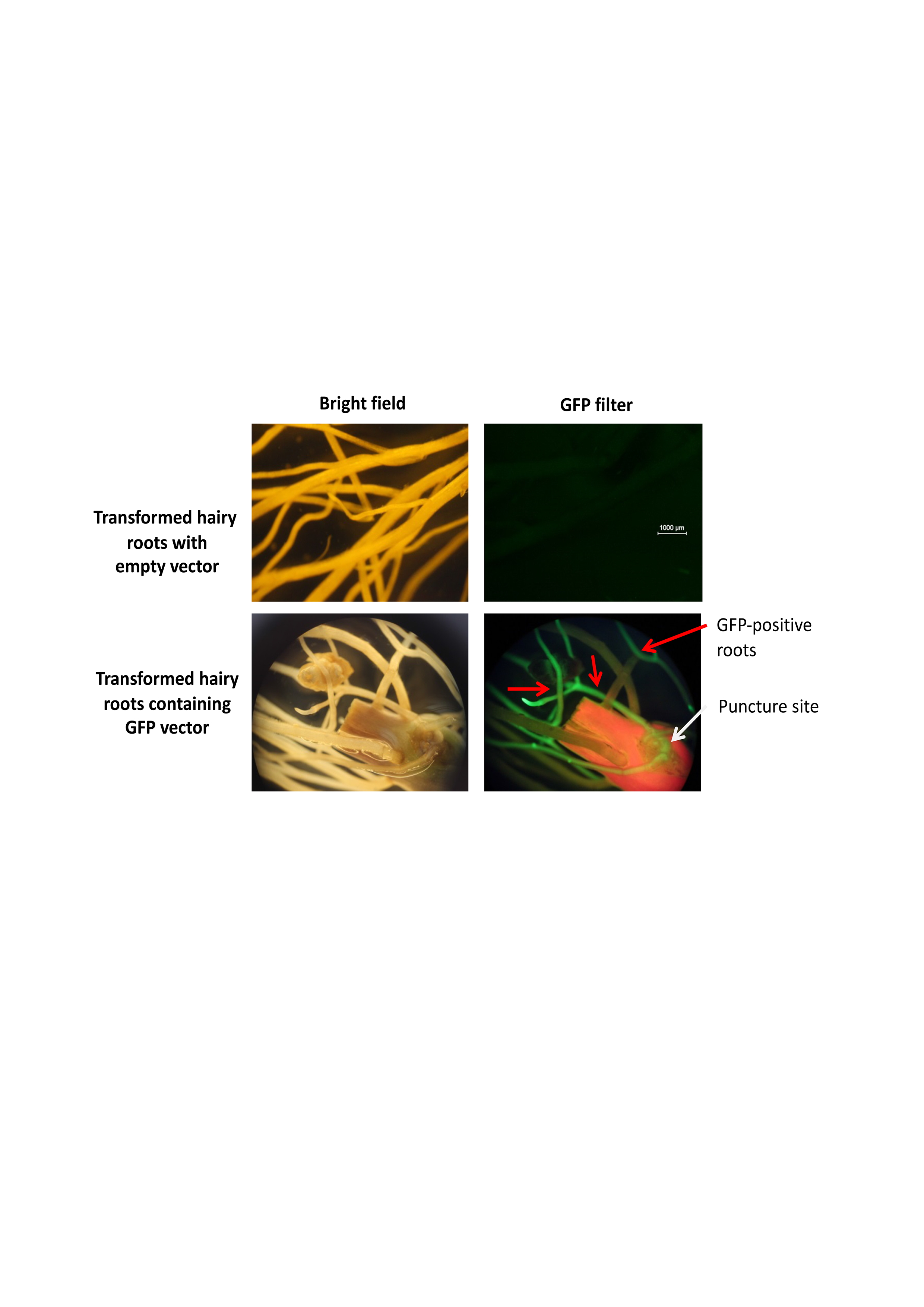

Supplement: Supplementary file 4 — Additional file 4: Figure S3. Transformation efficiency of soybean hairy roots assessed by a GFP reporter construct. Constructs containing Green Florescent Protein (GFP), under the control of 35S promoter or empty vectors, were introduced into Agrobacterium rhizogenes to induce hairy roots. Positive transgenic roots, indicated by red arrows, can easily be distinguished from non-transgenic roots by checking the GFP signal by microscopy. [file 13104_2019_4207_MOESM4_ESM.tif]
