# Supplementary material for: Mutagenesis of seed storage protein genes in Soybean using CRISPR/Cas9
Source: BMC Res Notes. 2019 Mar 27;12:176. doi: 10.1186/s13104-019-4207-2 (PMC6437971; doi:10.1186/s13104-019-4207-2)
Supplement: Supplementary file 5 — Additional file 5: Table S2. Summary of GFP-positive hairy roots at puncture sites. [file 13104_2019_4207_MOESM5_ESM.docx]

**Additional Table S2. Summary of GFP-positive hairy roots at puncture sites**

| **Plant ID** | **GFP-positive hairy roots per total**  **hairy roots at puncture site 1** | **% GFP-positive**  **roots at puncture**  **site 1** | **GFP-positive hairy roots per total**  **hairy roots at puncture site 2** | **% GFP-positive roots at puncture**  **site 2** |
| --- | --- | --- | --- | --- |
| 1 | 3/5 | 60% | 4/5 | 80% |
| 2 | 1/3 | 33% | 2/3 | 67% |
| 3 | 1/4 | 25% | 1/3 | 33% |
| 4 | 1/5 | 20% | 1/4 | 25% |
| 5 | 2/6 | 33% | 2/5 | 40% |
| 6 | 2/5 | 40% | 0/4 | 0% |
| 7 | 0/1 | 0% | 2/5 | 40% |
| 8 | 1/4 | 25% | 1/4 | 25% |
| 9 | 1/3 | 33% | 0/4 | 0% |
| 10 | 2/5 | 40% | 2/4 | 50% |
| 11 | 2/7 | 29% | 2/5 | 40% |
| 12 | 1/4 | 25% | 0/1 | 0% |
| 13 | 0/2 | 0% | 1/4 | 25% |
| 14 | 4/9 | 44% | 0/0 | 0% |
| 15 | 1/3 | 33% | 2/8 | 25% |
| 16 | 3/6 | 50% | 2/5 | 40% |
| 17 | 3/9 | 33% | 0/3 | 0% |
| 18 | 1/4 | 25% | 0/0 | 0% |
| 19 | 1/3 | 33% | 0/3 | 0% |
| 20 | 1/5 | 20% | 1/6 | 17% |
| 21 | 1/3 | 33% | 3/6 | 50% |
| 22 | 1/7 | 14% | 0/4 | 0% |
| 23 | 1/4 | 25% | 0/3 | 0% |
| 24 | 1/3 | 33% | 2/5 | 40% |
| 25 | 1/5 | 20% | 3/5 | 60% |
| 26 | 2/4 | 50% | 2/4 | 50% |
| 27 | 1/6 | 17% | 0/2 | 0% |
| 28 | 1/2 | 50% | 0/2 | 0% |
| 29 | 1/5 | 20% | 1/2 | 50% |
| 30 | 2/5 | 40% | 0/3 | 0% |
| 31 | 3/10 | 30% | 1/5 | 20% |
| 32 | 1/4 | 25% | 2/3 | 67% |
| 33 | 1/2 | 50% | 0/1 | 0% |
| 34 | 1/4 | 25% | 0/4 | 0% |
| 35 | 1/3 | 33% | 0/2 | 0% |
| 36 | 2/3 | 67% | 0/2 | 0% |
| 37 | 1/2 | 50% | 0/0 | 0% |
| 38 | 2/4 | 50% | 0/3 | 0% |
| 39 | 0/0 | 0.00 | 0/0 | 0% |
| 40 | 0/0 | 0.00 | 0/0 | 0% |
| 41 | 1/2 | 50% | 0/2 | 0% |
| 42 | 1/2 | 50% | 0/2 | 0% |
| 43 | 1/5 | 20% | 1/5 | 20% |
| 44 | 0/0 | 0.00 | 0/0 | 0% |
| 45 | 0/0 | 0.00 | 0/0 | 0% |
| 46 | 1/4 | 25% | 0/0 | 0% |
| 47 | 2/6 | 33% | 1/3 | 33% |
| 48 | 2/5 | 40% | 0/4 | 0% |
| 49 | 1/3 | 33% | 0/2 | 0% |
| 50 | 1/6 | 17% | 1/5 | 20% |
| 51 | 2/9 | 22% | 0/2 | 0% |
| 52 | 0/3 | 0% | 0/1 | 0% |
| 53 | 2/3 | 67% | 1/3 | 33% |
| 54 | 1/4 | 25% | 1/2 | 50% |
